# Greater severity of new onset asthma in allergic subjects who smoke: a 10-year longitudinal study

**DOI:** 10.1186/1465-9921-12-16

**Published:** 2011-01-24

**Authors:** Riccardo Polosa, Cristina Russo, Pasquale Caponnetto, Gaetano Bertino, Maria Sarvà, Tjana Antic, Stefania Mancuso, Wael K Al-Delaimy

**Affiliations:** 1 Dipartimento di biomedicina clinica e molecolare - S. Marta Hospital; azienda ospedaliero-universitaria O.V.E., Università di Catania, Catania, Italy; 2 Fondazione Salvatore Maugeri - U.O. neuroriabilitazione intensiva, Mistretta (Messina), Italy; 3 Department of family and preventive medicine, University of California, San Diego, USA

## Abstract

**Background:**

Little is known about the association between cigarette smoking and asthma severity. We assessed smoking as a determinant of disease severity and control in a cohort of clinic-referred allergic subjects who developed new onset asthma.

**Methods:**

Allergic rhinitis subjects with no asthma (n = 371) were followed-up for 10 years and routinely examined for asthma diagnosis. In those who developed asthma (n = 152), clinical severity and levels of asthma control were determined. Among these subjects, 74 (48.7%) were current smokers, 17 (11.2%) former smokers, and 61 (40.1%) never smokers.

**Results:**

When comparing current or past smokers to never smokers they had a higher risk of severe asthma in the univariate analysis, which became non-significant in the multivariate analysis. On the other hand, the categories of pack-years were significantly related to severe asthma in a dose response relationship in both the univariate and multivariate analysis: compared to 0 pack years, those who smoked 1-10 pack-years had an OR(95% CI) of 1.47(0.46-4.68), those who smoked 11-20 pack-years had an OR of 2.85(1.09-7.46) and those who smoked more than 20 pack-years had an OR of 5.59(1.44-21.67) to develop more severe asthma. Smokers with asthma were also more likely to have uncontrolled disease. A significant dose-response relationship was observed for pack-years and uncontrolled asthma. Compared to 0 pack years, those who smoked 1-10 pack-years had an OR of 5.51(1.73-17.54) and those who smoked more than 10 pack-years had an OR of 13.38(4.57-39.19) to have uncontrolled asthma.

**Conclusions:**

The current findings support the hypothesis that cigarette smoking is an important predictor of asthma severity and poor asthma control.

## Background

Beside the notion that accelerated decline in lung function over time is present in asthmatic individuals who smoke [1-3], adults and older children with asthma who are active smokers have also more severe symptoms and worse asthma-specific quality of life compared to asthmatic non-smokers [4-6]. Asthma mortality is greater among asthmatics who smoke cigarettes compared to asthmatics who do not smoke [7,8]. In addition, asthmatic patients who smoke appear to have a reduced therapeutic response to inhaled and oral corticosteroids [9-11]. Recent research has identified genes associated with increased risk for asthma in the presence of tobacco smoke exposure [12] and demonstrated that cigarette smoking is an important independent risk factor for new onset asthma in allergic individuals [13].

Increased hospital admission rates seen in some countries are not simply due to the overall increased prevalence of asthma, but are also likely to be related to a greater severity of the disease [14]. Although factors such as gender, atopy, duration of asthma, bronchial hyperresponsiveness (BHR) and frequent asthma exacerbations appear to be important determinants of the severe asthma phenotype [15], the association between common modifiable risk factors such as cigarette smoking and asthma severity has received surprisingly little attention.

Limitations of the few studies addressing the relationship between cigarette smoking and asthma severity and asthma control included the reliance on cross-sectional and case-control study design, the use of poor measures of tobacco smoke exposure, or the absence of agreed criteria on level of asthma severity/control. Until recently, the global initiative for asthma (GINA) [16] has been widely used and accepted as a comprehensive and valid measure of asthma severity in adults. Yet, severity is not a stable feature of asthma, and the classification of an individual by disease severity suggests a static feature, which is clearly not the case in everyday clinical asthma where the level of severity varies in relation to the amount of antiasthma medication taken. These considerations induced the GINA faculty and the US colleagues of the national asthma education prevention program (NAEPP) to modify in recent years their views about asthma severity and to promote the more clinically informative concept of asthma control [17,18]. Nonetheless, the previous GINA 2002 classification of asthma by severity into intermittent, mild persistent asthma, moderate persistent and severe persistent is still recommended for research purposes [19], as it is likely to better reflect the intrinsic characteristic of an asthma phenotype in the absence of pharmacological confounders (i.e. regular antiasthma therapies).

Aim of this study was to investigate whether cigarette smoking could be a determinant of disease severity and poor asthma control in a cohort of clinic-referred allergic subjects who developed new onset asthma. From the case notes of a relatively large clinic cohort of adult subjects with allergic rhinitis followed-up for 10 years, we calculated the severity class (GINA 2002) by reviewing their personal medical notes at the time of their first documentation of asthma symptoms and/or abnormal lung function. On the other hand, levels of asthma control were determined at the end of the follow-up according to the classification set in the third revision of the expert panel report for the national asthma education prevention program (NAEPP EPR3). We then investigated the importance of cigarette smoking as a determinant of asthma severity (using the GINA 2002 grading) and asthma control (adopting the NAEPP EPR3 criteria).

## Material and methods

### Study population

Our initial study population sample consisted of a cohort of 371 clinic-referred non-asthmatic adults with allergic rhinitis for whom all explanatory variables, asthma status at follow-up, and smoking history were available. Full details of this population sample have been previously described [13-20]. In brief, medical records of subjects with a diagnosis of allergic rhinitis in the age range 18-40 yrs and not diagnosed with asthma at the time of their first visit at the outpatient allergy clinic of the University of Catania (Sicily) in the period between January 1990 and December 1991 were reviewed. Our standardized diagnostic protocol at the time of their first visit consisted of case history, clinical examination, spirometry, and skin tests to most common aeroallergens including *Parietaria judaic*a, *Dermatophagoides pteronyssinu*s, *Dermatophagoides farinae, Olea europea*, grass pollen, orchard, cypressus, alternaria, perennial rye, and cat allergen. Details about their smoking history were collected in addition to questions on the family history for atopic disease and second-hand smoke exposure history. The diagnostic criteria used for allergic rhinitis were those defined by the joint task force on practice parameters in allergy, asthma and immunology [21]. Subjects with allergic rhinitis simply requiring symptomatic over the counter drugs, such as topical decongestants, intranasal sodium cromoglycate, and/or oral antihistamines throughout the whole duration of the study were included. Subjects using nasal corticosteroids for more than 6 weeks/year were not included. Subjects were excluded from the study cohort if there was a past or present history of asthma, previous asthma symptoms or asthma medication intake, and/or abnormal spirometric values at the time of their first visit. The criteria used for the diagnosis of asthma during follow up were those based on the recommendations established by the american thoracic society (ATS) [22]. Specifically, diagnosis of asthma had to be documented in at least three consecutive control visits from the time of initial referral. All subjects were born and residing in the same district (province of Catania - Sicily).

**Figure 1:**
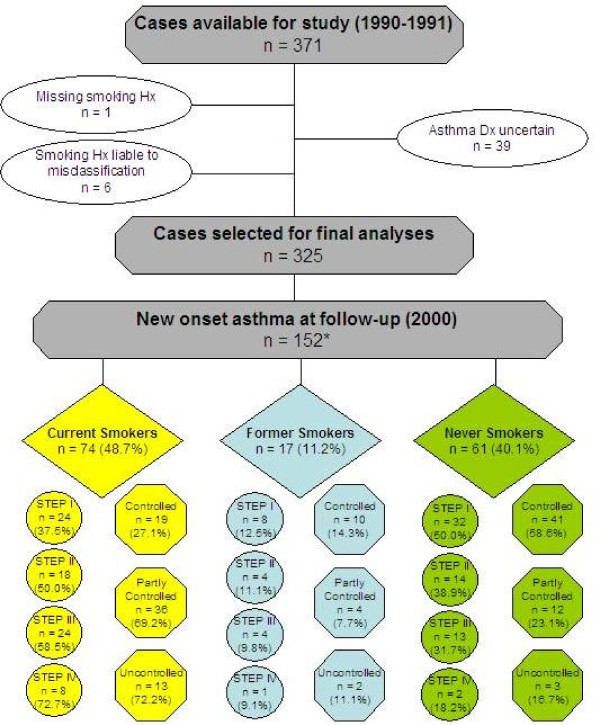
**Study flow chart**. Medical records of cases with allergic rhinitis in the period between January 1990 and December 1991 were reviewed. To be included in the study cases had to be between the ages of 18 and 40 years and not diagnosed with asthma. Out of the initial 371 cases, complete study outcomes variables were available from 325 subjects. A diagnosis of asthma could be established at final review (from January to April 2000) in 152 subjects. Among these subjects, 74 (48.7%) were current smokers, 17 (11.2%) former smokers, and 61 (40.1%) never smokers. Clinical severity class (circles) and level of asthma control (octagons) were then computed for each categorized smoking status. * At final review, asthma control data could not be calculated in 12 of those with new onset asthma due to lack of information about exacerbations.

Out of the initial 371 cases, study outcomes variables were available from 325 subjects (Figure 1). Data from 46 subjects were excluded from analyses for several reasons: a diagnosis of asthma could not be established with confidence (n = 39); occasional smokers (with a pack/yrs < 1) at baseline that never became regular smokers (n = 6); smoking history was missing (n = 1). At the final control visit, in the period from January 2000 to April 2000, a total of 152 subjects were found to have developed new onset asthma. Among these subjects, 74 (48.7%) were current smokers, 17 (11.2%) former smokers, and 61 (40.1%) never smokers. In 12 of those with new onset asthma at the final follow-up visit, asthma control data could not be calculated due to lack of information about exacerbations and were not included in the analyses relevant to that outcome. The study protocol was approved by the local institutional ethics and review board.

### Study design, explanatory and outcome variables

Subjects selected at baseline (Jan1990- Dec1991) were evaluated for possible asthma symptoms throughout the study and were then invited for a control visit in the period from January to April 2000 to complete a questionnaire on respiratory/allergic conditions (modified from the ISAAC core questions - 23), and to review their smoking history.

If a diagnosis of asthma was confirmed, clinical severity class and level of asthma control were computed. Each subject's GINA 2002 severity class [16] was calculated by reviewing their personal medical documentation at the time of their first report of asthma symptoms and/or abnormal lung function. Respiratory symptoms (day-time and night-time cough, wheezing, or breathlessness) and lung function data were noted. Based on this classification, asthma severity is graded in to four steps: Step I - intermittent asthma; Step II - mild persistent asthma; Step III: moderate persistent asthma; Step IV: severe persistent asthma.

Levels of asthma control were determined at the final follow up visit. According to NAEPP EPR3 [18], asthma control can be categorized into three levels based on frequency and intensity of current (within the previous 2 weeks) symptoms, use of beta2-agonist to treat symptoms, lung function data and number of exacerbations (in the previous 12 months): Level 1 - well controlled asthma; Level 2 - partially controlled asthma; and Level 3 - poorly controlled asthma.

Correlation between severity and control was r = 0.26 (p = 0.002). A cross table between severity and control shows that Level 1 of control had 50,0% of GINA Step I and only 1,4% of GINA Step IV, whereas Level 3 of control had 16,7% of GINA Step I, but 11,1% of GINA Step IV.

### Statistical Methods

The t-test and chi-squared test (or Fisher's exact test for variables with less than 5 frequencies per category) were used to determine the level of significance of variables according to smoking status.

Logistic regression was used to assess the association of asthma severity risk factors at baseline with level of asthma severity and asthma control as outcomes. These risk factors in the model were: age (years), gender (male, female), or presence of family history of atopy (yes, no), dichotomous smoking status (smoking vs non-smoking). One of the following smoking variables were used separately for each model adjusting for the above variables: passive smoking (yes, no), categorized smoking status (never smokers, former smokers, current smokers), and pack-years categories (0, 1-10, 11-20, and 21+ pack-years). Four separate models were run according to the tobacco exposure variables of model 1: passive smoking among never smokers when current and past smokers excluded; model 2: current or past smokers compared to never smokers; model 3: pack-years categories when past smokers and those smokers at baseline who quit smoking at the end of follow up were excluded; model 4: smoking status of never smokers, past and current smokers, excluding those who quit smoking at the end of follow up. For the asthma control outcome, family history of atopy was excluded because there was an exactly similar percentage of cases with family history of atopy in both categories of asthma control. Because there were smaller number of asthma control outcomes, the category of more than 20 pack-years contained only 4 subjects and the category of 11-20 pack-years contained only 5 subjects with lower asthma control, they were therefore combined into one category of more than 10 pack-years in the logistic regression models.

## Results

The demographic characteristics of the study population across the 3 smoking categories are described in Table 1 (see also Figure 1). Current smokers were slightly older, more likely to be females than males, and more likely to develop severe asthma and suboptimal control of their disease. Out of a total study population of 152 subjects with new onset asthma, 64 were classified as GINA Step I, 36 as GINA Step II, 41 as GINA Step III, and 11 as GINA Step IV. Due to the small numbers in some GINA categories, we combined together class severity Step I + II, and Step III + IV. Control level could not be classified in 12 subjects due to lack of information about exacerbations. Therefore, out of a total study population of 140 subjects, 70 were classified as being "controlled", 52 as being "partly controlled", and 18 as being "uncontrolled". Due to the small numbers in the "uncontrolled" category, it was combined with the "partly controlled" category for the statistical analyses.

**Table 1:** Characteristics of the study population developing new onset asthma (N = 152) in relation to smoking status at baseline and during follow-up

**Characteristics of the study population at baseline**			
**Variables**	**Never Smokers N = 61**	**Former Smokers N = 17**	**Current Smokers N = 74**
Age, mean yr (±SD)	28.5 (6.4)	28.6 (7.2)	30.7 (5.4)
Sex			
*Female*	52.5%	47.1%	60.8%
*Male*	47.5%	52.9%	39.2%
Exposure to passive smoking	65.6%	70.6%	62.2%
Family Hx of atopy	75.4%	70.6%	67.6%
Duration of rhinitis, mean yr (±SD)	8.4 (5.1)	10.2 (7.9)	9.0 (5.8)
FEV1 (%predicted), mean (±SD)	96.8 (11.1)	102.5 (18.4)	99.0 (10.2)
Sensitizations			
+ve skin test to *Parietaria*	63.2%	59.0%	65.9%
+ve skin test to *HDM*	34.5%	37.1%	33.0%
+ve skin test to *Olea*	27.5%	24.9%	25.5%
+ve skin test to *Grass pollen*	22.0%	25.8%	24.7%
+ve skin test to *Cat*	8.5%	7.7%	9.0%
Drugs for rhinitis symptoms			
*Oral antihistamines*	58.3%	54.8%	54.0%
*Topical decongestants*	37.0%	35.9%	33.3%
*Nasal steroids (<6 wks/yr)*	20.5%	17.0%	19.2%
**Characteristics of the study population during follow-up**			
Drugs for asthma symptoms			
*SABA*	85.2%	81.9%	87.5%
*ICS*	27.6%	30.1%	35.8%
*Others*	15.3%	17.2%	18.8%
Asthma symptoms/day			
*Weekly basis or less*	76.2%	71.7%	47.8%
*Daily/continous*	23.8%	28.3%	52.2%
Asthma symptoms/night			
*Monthly basis or less*	85.1%	81.5%	62.2%
*Weekly/frequent*	14.9%	18.5%	37.8%
SABA to treat symptoms			
≤ *2 days/wk*	69.4%	58.0%	26.1%
*> 2 days/wk*	21.4%	31.1%	51.4%
*Daily use*	9.2%	10.9%	22.5%
Exacerbations (in the past yr)			
*0-1/yr)*	76.3%	70.8%	56.5%
≥ *2/yr*	23.7%	29.2%	43.5%
Asthma Severity (N = 152)			
*GINA Step I*	50.0%	12.5%	37.5%
*GINA Step II*	38.9%	11.1%	50.0%
*GINA Step III*	31.7%	9.8%	58.5%
*GINA Step IV*	18.2%	9.1%	72.7%
Asthma Control (N = 140)			
*Controlled*	58.6%	14.3%	27.1%
*Partly controlled*	23.1%	7.7%	69.2%
*Uncontrolled*	16.7%	11.1%	72.2%

HDM, house dust mite; SABA, short acting β2 agonists, ICS, inhaled corticosteroids

**Figure 2:**
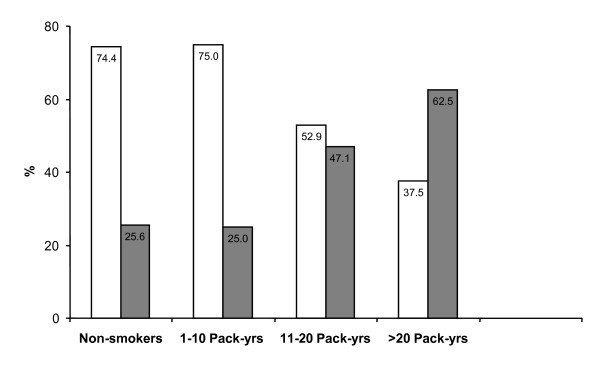
**Percentage of subjects with less severe (GINA Step I and II; white bars) and more severe (GINA Step III and IV; grey bars) forms of asthma among the non-smokers and those who smoked**. Estimation of the amount and duration of smoking exposure was established by calculating pack-years. Smokers were therefore categorized by incremental pack-years.

Smokers had a higher risk of severe asthma with a significant dose-response relationship (Chisq = 11.63, p = 0.009). Our results indicate that 25.6% (20/78) of non smokers developed a severe form of asthma (GINA Steps III + IV), and similarly only 25% (6/24) of those who smoked 1-10 pack years developed a severe form of the disease compared to 47.1% (16/34) of those who smoked 11-20 pack years developed a severe form of asthma and even a higher percentage (62.5%; 10/16) of severe asthma cases were identified in those who smoked more than 20 pack years (Figure 2).

**Figure 3:**
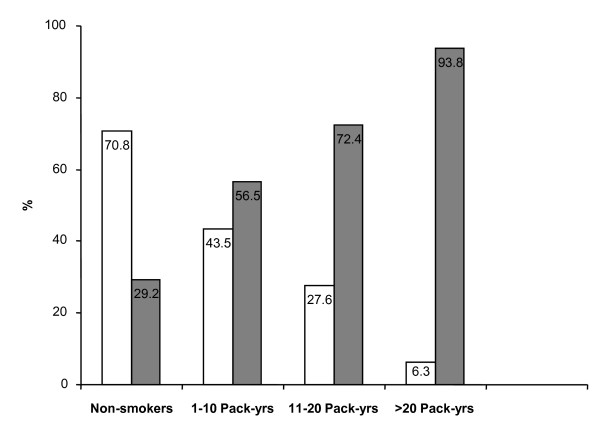
**Percentage of subjects with optimal ("Controlled"; white bars) and suboptimal ("Partly Controlled" and "Uncontrolled"; grey bars) asthma control among the non-smokers and those who smoked**. Estimation of the amount and duration of smoking exposure was established by calculating pack-years. Smokers were therefore categorized by incremental pack-years.

We have also found that pack-years use was significantly associated with a progressive loss of asthma control (Chisq = 30.97, p < 0.0001). Whereas only 29.2% (21/72) of non-smokers were categorized as having partly controlled or uncontrolled asthma, 56.5% (13/23) of those who smoked 1-10 pack-years, 72.4% (21/29) of those who smoked 11-20 pack-years, and 93.8% (15/16) of those who smoked more than 10 pack-years developed a partly controlled or uncontrolled form of the disease (Figure 3).

Results from the univariate and multivariate analyses of measures of asthma severity are presented in Table 2. When comparing current or past smokers to never smokers, they had a higher risk of severe asthma in the univariate analysis but became non-significant in the multivariate analysis. On the other hand the categories of pack years were significantly related to severe asthma in a dose-response relationship in both the univariate and multivariate analysis. In the multivariate analyses models we found that, compared to those who did not smoke (0 pack-years), those who smoked 11-20 pack years had an odds ratio of 2.85 (95% CI 1.09-7.46), and those who smoked more than 20 pack years had an odds ratio of 5.59 (95% CI 1.44-21.67). Similarly current smokers were significantly related to have more severe asthma compared to never smokers 2.78 (95% CI 1.28-6.08) after adjustment of the other covariates.

**Table 2:** Univariate and multivariate odds ratio of asthma severity (GINA Step III and IV Combined) According to asthma risk factors and smoking variables

**Variables**	**Univariate Odds Ratio (95% CI)**	**P value**	**Multivariate Odds Ratio (95% CI)**	**P value**
**Gender**				
Female	1.00			
Male	1.14 (0.58-2.23)	P = 0.71		
**Age**	1.07 (1.01-1.31)	P = 0.027		
**Family Hx of Atopy**	0.76 (0.37-1.58)	P = 0.46		
**Model 1 (n = 61)**	1.07 (0.31-3.66)	P = 0.92	1.19 (0.32-4.50)	P = 0.79
Passive Smoking				
**Model 2 (n = 152)**		P = 0.043		
Current or Former Smoker	2.10 (1.03-4.31)		1.93 (0.93-4.01)	P = 0.08
**Model 3 (n = 125)**				
Packs per Year				
Never Smoker 0	1.00		1.00	
1-10 packs 1	1.31 (0.43-4.03)	P = 0.63	1.47 (0.46-4.68)	P = 0.52
11-20 packs 2	3.51 (1.39-8.83)	P = 0.008	2.85 (1.09-7.46)	P = 0.032
>20 packs 3	7.67 (2.09-28.07)	P = 0.002	5.59 (1.44-21.67)	P = 0.013
**Model 4 (n = 142)**				
Smoking Status				
Never Smoker 0	1.00		1.00	
Former Smoker 1	1.28 (0.39-4.22)	P = 0.69	1.26 (0.37-4.24)	P = 0.79
Current Smoker 2	3.07 (1.43-6.57)	P = 0.004	2.78 (1.28-6.08)	P = 0.009

Model 1: Reference group are nonsmokers not exposed to passive smoking. Past and current smokers excluded.

Model 2: Reference group are nonsmokers. No exclusions.

Model 3: Past smokers and those who quit during follow up excluded.

Model 4: Those who quit smoking during follow up excluded

Results from the univariate and multivariate analyses of measures of asthma control are presented in Table 3. When comparing current or past smokers to never smokers, they had a higher risk of poor asthma control in both univariate and multivariate analyses. Likewise, categories of pack-years were significantly related to the level of asthma control in a dose-response relationship in both the univariate and multivariate analysis. In the multivariate analyses models we found that, compared to those who did not smoke (0 pack-years), those who smoked 1-10 pack years had an odds ratio of 5.51 (95% CI 1.73-17.54), and those who smoked more than 10 pack years had an odds ratio of 13.38 (95% CI 4.57-39.19) to have uncontrolled asthma. Similarly current smokers were significantly more likely to have poorly controlled asthma compared to never smokers 9.54 (95% CI 3.98-22.88) after adjustment of the other covariates.

**Table 3:** Univariate and multivariate odds ratio of asthma control (Partly controlled and uncontrolled combined)* according to asthma risk factors and smoking variables

**Variables**	**Univariate Odds Ratio (95% CI)**	**P value**	**Multivariate Odds Ratio (95% CI)**	**P value**
**Gender**				
Female	1.00			
Male	1.19 (0.61-2.32)	P = 0.61		
**Age**	1.06 (1.00-1.12)	P = 0.06		
**Model 1 (n = 56)**	0.53 (0.16-1.78)	P = 0.30	0.53 (0.16-1.81)	P = 0.31
Passive Smoking				
**Model 2 (n = 140)**				
Current or Former	5.18 (2.47-10.90)	P < 0.0001	5.18 (2.43-11.02)	P < 0.0001
Smoker				
**Model 3 (n = 114)**				
Packs per Year				
Never Smoker 0	1.00		1.00	
1-10 packs 1	4.69 (1.55-14.13)	P = 0.006	5.51 (1.73-17.54)	P = 0.004
>10 packs 2	15.03 (5.25-43.03)	P < 0.0001	13.38 (4.57-39.19)	P < 0.0001
**Model 4 (n = 130)**				
Smoking Status				
Never Smoker 0	1.00		1.00	
Former Smoker 1	1.64 (0.51-5.30)	P = 0.41	1.70 (0.52-5.58)	P = 0.38
Current Smoker 2	9.46 (4.03-22.24)	P = <0.0001	9.54 (3.98-22.88)	P < 0.0001

Model 1: Reference group are nonsmokers not exposed to passive smoking. Past and current smokers excluded.

Model 2: Reference group are nonsmokers. No exclusions.

Model 3: Past smokers and those who quit during follow up excluded.

Model 4: Those who quit smoking during follow up excluded

* Uncontrolled combined with Partly Controlled because only 18 individuals were in the Uncontrolled category. Now we have 70 individuals in the Controlled category and 70 individuals in the Uncontrolled and Partly Controlled categories combined.

## Discussion

Our study is the first clinical cohort establishing the importance of cigarette smoking as a determinant of disease severity and control in allergic subjects who developed new onset asthma. Smoking status and smoking duration were markedly related in a dose-dependent fashion to the level of asthma severity and to poor asthma control. The demonstration of strong association and clear-cut dose-response relationship of smoking with asthma severity and control is in support of causality.

Previous surveys have used cross-sectional and case-control design, employed ill-defined asthma severity criteria, and mostly relied on questionnaires for the documentation of asthma symptoms and smoking status [4-6,24,25]. Relying on questionnaires for the documentation of asthma symptoms may be unsatisfactory and cumulative exposure of tobacco measured by pack-years is more important than plain smoking status. Moreover, the possibility that treatment modalities (especially regular topical corticosteroids) might have altered the severity of the disease in the previous studies cannot be excluded. Lastly, our well characterized clinic cohort of allergic subjects at high risk for incident asthma represents an exclusive experimental model in which the effect of a common environmental risk factor (i.e. cigarette smoking) can be studied in relation to the progression of the natural history of the disease and circumvents the methodological limitations of previous surveys that have used cross-sectional and case-control design.

Using the GINA severity classification, data from our analyses show that smoking status and smoking duration are markedly related in a dose-dependent fashion to more severe asthma. The strongest association with more severe disease being observed in those who smoked more than 20 pack-years. Our findings largely agree with what has been illustrated in previous surveys [4-6,24,25], but here we show for the first time that disease severity is associated with increased pack-years in a dose-response relationship.

Our cohort study of non-asthmatic adults with allergic rhinitis and followed up for 10 years shows that smoking can predict not just asthma incidence [13], but also severity and level of control of the disease (this paper). We do not know the exact mechanism by which asthmatics who smoke have a more severe form of the disease, but it is likely that the inherent biologic intensity of the asthmatic inflammatory process is amplified by active smoking. Cigarette smoking may induce a neutrophil-predominant inflammation of the airways [26,27], which may render patients less responsive to asthma treatment [9-11]. Moreover, persistent exposure to cigarette smoke not only enhances allergic Th2-driven inflammation [28], but also Th1-mediated inflammatory responses [26,29]. Given that a mixed Th1/Th2 inflammatory response is a key event in the process of developing a more severe asthma phenotype [15], development of a more severe disease may be anticipated in those allergic individuals who smoke regularly.

Smoking status and smoking duration are also markedly related in a dose-dependent fashion to poor asthma control, the strongest association with poor controlled disease being observed in those who smoked more than 10 pack-years. This is in agreement with recent population-based surveys of smoking status in asthma from Switzerland (30), UK (31), France [32] and United States [33]. The reason for asthmatics who smoke to have uncontrolled disease is not clear, but behavioral factors such as non-adherence and poor inhaler technique may play a role [34,35]. In particular, non-adherence with antiasthma medications is common in asthmatic patients who are smokers [36]. Additionally, poor asthma control can be due to the reduced therapeutic response to inhaled and oral corticosteroids in asthmatics who smoke [9-11]. Another potential reason for apparent poor asthma control among smokers is misdiagnosis of chronic obstructive pulmonary disease (COPD) as asthma. Research suggests that up to one third of smokers over the age of forty with an asthma diagnosis may in fact have COPD [37]. Although we cannot rule out completely a diagnosis of concomitant COPD in those who smoked, misdiagnosis of COPD in our study is unlikely as a result of the relatively young age of the study population entry criteria who had to be between the ages of 18 and 40 years.

A possible limitation of our study includes relying on medical records for the selection of the study subjects. However, all these subjects were examined and carefully diagnosed and documented in the clinic by experienced allergy specialists. Pack-years is a crude estimate of the amount and duration of smoking exposure, but this is universally used to address duration of exposure to tobacco smoke [38], and in the present study allows us to demonstrate clear dose-related associations with disease severity and control. Another possible weakness of our study includes relying on a relatively small sample size to run multiple linear regression analyses on four class of asthma severity and three category of disease control for current smokers, former smokers, and never smokers. We minimized this problem by combining together class severity (Step I with II, and Step III with IV) and category of control ("uncontrolled" with "partly controlled"). Lastly, incomplete assessment of other important factors may limit our ability to define the relative importance of the key determinants of asthma control, as we did not collect information on compliance, socio-economical status, and education.

Our study has the advantage of the rigorous clinical assessment of asthma symptoms, medication use and lung function during the follow up visit at the same clinic. This is a substantial advancement compared with previous work in which self-report documentation of asthma symptoms, lack of objective measures for the diagnosis of asthma, and the cross-sectional design represented a severe limitation. Also, the fact that the all subjects examined were atopic (mostly sensitized to *Parietaria judaica*- the most prevalent allergen in Sicily) contributed to an important reduction in confounding factors for asthma severity/control. Furthermore, the possibility that regular nasal corticosteroids might have influenced study outcomes was addressed by excluding subjects using nasal corticosteroids for more than 6 weeks/year. Lastly, by examining asthma at ages when chronic obstructive pulmonary disease (COPD) is not prevalent, we have minimized this important confounders of poor asthma control.

The negative impact of smoking on asthma severity and control appears to be at least partially reversible in our study, as patients who had quit smoking reported significantly less severity and better asthma control than current smokers. This may have profound implications for clinical practice. If a modifiable determinant of asthma severity and poor control such as smoking can be easily identified in routine practice, it should be addressed in order to reduce asthma severity and to improve asthma control. Indeed, smoking cessation is associated with improvements in asthma symptoms, lung function quality of life scores, and BHR [39-41] and most recent GINA guidelines recommend that smoking cessation should be an integral part of asthma treatment strategy.

## Competing interests

The authors declare that they have no competing interests.

## Authors' contributions

RP carried out the design and coordination of the study, gathered and interpreted the data, drafted and finalized the manuscript. WA participated in the design of the study and performed the statistical analysis. CR, PC, GB, MS, TA and SM. were involved in the coordination and design of the study, helped to interpret the data and the critically revised the manuscript. All authors read and approved the final version of the manuscript.
